# Examination of Self-Esteem, Body Image, Eating Attitudes and Cardiorespiratory Performance in Adolescents

**DOI:** 10.3390/ijerph182413172

**Published:** 2021-12-14

**Authors:** Peter Petrovics, Alexandra Nagy, Barbara Sandor, Anita Palfi, Zsolt Szekeres, Kalman Toth, Eszter Szabados

**Affiliations:** 1 Institute of Physical Education and Sport Sciences, Faculty of Sciences, University of Pecs, H-7624 Pecs, Hungary; petropet@gamma.ttk.pte.hu; 2 State Hospital for Cardiology, H-8230 Balatonfüred, Hungary; nagy.alexandra@szivkorhaz.hu; 3 Division of Preventive Cardiology and Rehabilitation, 1st Department of Medicine, Medical School, University of Pecs, H-7623 Pecs, Hungary; sandor.barbara@pte.hu (B.S.); palfi.anita@pte.hu (A.P.); szekeres.zsolt@pte.hu (Z.S.); 4 Division of Cardiology, 1st Department of Medicine, Medical School, University of Pecs, H-7624 Pecs, Hungary; toth.kalman@pte.hu

**Keywords:** adolescent, self-esteem, body image, eating attitudes, cardiorespiratory performance

## Abstract

Self-esteem, body image and eating attitudes are important characteristics regarding adolescent mental health. In our present work, we aimed to investigate these psychological items in adolescent boys and girls examining gender differences and correlations with the BMI-for-age and cardiorespiratory performance. 374 students (209 girls with an average age of 16.4 ± 1.08 years, and 165 boys with an average age of 16.5 ± 1.03 years) underwent investigation using the Rosenberg self-esteem scale, EAT-26 and BAT questionnaires. The BMI-for-age was calculated with BMI growth charts and the cardiorespiratory performance was measured with the 20 m shuttle run test. Our results showed that adolescent girls scored lower self-esteem and higher values for BAT and each scale of eating behaviors, such as uncontrolled eating, cognitive restraints and emotional eating compared to boys despite the fact, that obesity and overweight were more common among boys. No significant correlation was found between BMI and psychological test results in either boys or girls, however, subjective body shape and gender predicted self-esteem and BAT scores and the cognitive restraints in the eating attitudes. Uncontrolled and emotional eating were primarily influenced by gender, in which BMI played only a weaker role. Cardiorespiratory performance was positively associated with self-esteem and body image among boys, and it had a negative correlation regarding BMI in both genders.

## 1. Introduction

Adolescence represents a sensitive period in which significant physical and psychological changes occur. Psychological characteristics, such as self-esteem and positive well-being may play a pivotal role in physical health. Better mental health during adolescence predicts better general health and fewer risky health behaviors during young adulthood [1]. Self-esteem can be defined as a subjective self-assessment based on prior learning and experiences and reflects how an individual sees and evaluates oneself [2]. Lower self-esteem can be a causal factor for depression, anxiety, eating disorders, high-risk behaviors, and social functioning [3]. In adolescence, a decline in the level of self-esteem can be observed [4], especially among girls [5]. Additionally, overweight and obese adolescents frequently experience low self-esteem. Interestingly, authors in a large meta-analysis found no improvement in self-esteem in obese or overweight adolescents following significant weight loss [6]. Good self-esteem facilitates engaging in health-promoting behaviors and maintaining good health [7]. Inversely, inadequate self-esteem may result in risk behaviors [8] and has been related to undesirable eating behaviors, an inactive lifestyle, diminished performance in areas of academics and elevated risk in developing symptoms associated with depression [9].

Body image, another remarkable psychological factor in adolescence, was defined as ‘a person’s perceptions, thoughts, and feelings about his or her body’ by Grogan [10]. Body dissatisfaction occurs when views of the body are deemed negative and the body image represents a discrepancy between the individual’s actual and ideal body [11]. Previous research suggests that women and adolescent girls experience higher levels of body dissatisfaction and disturbed eating patterns than when compared with their male counterparts [12,13]. Women are more likely than men to describe themselves as fat or weigh themselves more often and they are also generally more dissatisfied with their physical appearance than are men [14]. The body dissatisfaction that is presented by adults can already be found in adolescents [15]. It is estimated that nearly 50% of adolescent girls are dissatisfied with their bodies [16]. Body dissatisfaction may lead to adverse physical and mental health consequences, including depression, anxiety [17], low self-esteem [18], and eating disorders [19,20].

Unhealthy eating attitudes and behaviors are quite common among young people, and overweight and obesity imply a particularly high risk of developing such behaviors [21,22]. The three most frequently studied areas regarding eating attitudes are uncontrolled eating (UE), cognitive restraint (CR), and emotional eating (EE). UE refers to an inclination to overeat, including the feeling of losing control [23]. CR suggests a tendency to consciously restrict food intake rather than using physiological cues (i.e., hunger and satiety) as regulators of eating [24]. Finally, emotional eaters tend to eat in response to emotional triggers rather than real physiological needs [25]. Emotional eating also implies or predicts weight gain [26] and difficulty of losing weight [27]. Compared to non-emotional eaters, emotional eaters are more prone to consume sweet and high-fat foods [28] and are more likely to eat in response to stressors [29]. Many eating disorders appear to start soon after puberty and persist through secondary school years [30]. However, most of these studies focus on girls and women and less attention is paid to boys and men, albeit eating disorders in men are increasing [31]. In the current study, we focus on disordered eating attitudes that do not reach clinical levels but might be predictive for developing an eating disorder.

Cardiorespiratory performance, which is a good indicator of overall physical health, can be defined as the capacity of the cardiovascular and respiratory systems to provide an adequate amount of oxygen during prolonged or strenuous exercise. Low cardiorespiratory fitness in children and adolescents has been associated with increased body fatness [32,33], hypertension [32,34] increased risk of metabolic syndrome [35,36] and depression [37]. Improvement in cardiorespiratory fitness has a positive effect in combating depression, anxiety, mood status, self-esteem, and is seemingly associated with higher performance in education [38]. In a recent clinical trial, cardiorespiratory fitness was independently and positively associated with self-related health in children (8–11.9 years age) and adolescents (12–17.9 years age) at baseline, including a two-year follow-up [39].

In our present work, we aimed to investigate the correlations depicted in areas of self-esteem, body image, eating attitudes and BMI-for-age and cardiorespiratory performance among adolescent boys and girls, since the correlations between these psychological and physical items have not been unequivocally described in previous studies. Furthermore, in the current study, we attempted to compare whether there is a difference in gender concerning our physical and psychological variables.

## 2. Materials and Methods

### 2.1. Study Participants

A total of 374 students from fourth-grade high school classes were enrolled in our prospective study (209 girls with an average age of 16.4 ± 1.08 years, and 165 boys with an average age of 16.5 ± 1.03 years). Written informed consent was obtained from all the participating adolescents for the measurements and the anonymous use of data purely for scientific purposes. Parents were also asked to sign the form authorizing the measurements and data handling. The study was approved by the Regional Ethics Committee of the University of Pecs (7522-PTE 2018) and was performed in 2018.

### 2.2. Measurements

Bodyweight was measured to the nearest 0.1 kg using an electronic digital body weighing scale, and height was measured to the nearest 0.1 cm using a manual height board. To screen for overweight and obesity, body mass index (BMI) and sex- and age-specific BMI-for-age were calculated using the BMI-for-age CDC growth charts [40]. Adolescents with a BMI-for-age ≥ 95th percentile were considered obese, between the 85th and 95th percentiles were classified as overweight, and with a BMI-for-age of <85th percentile were considered normal. The cut-off value for underweight was less than the 5th percentile of the BMI-for-age.

In consideration of the assessment regarding the cardiorespiratory performance of the adolescents, a 20 m shuttle run test was used. The 20 m shuttle run test is the most widely used field-based assessment of cardiorespiratory fitness [41].

This test involves continuously running between two lines, 20 m apart, in time, to recorded beeps. The participants are first positioned behind one of the lines facing the second line and begin running when instructed by the recording. The participant continues running between the two lines, turning when signaled by the recorded beeps. A sound indicates an increase in speed in minute intervals. A participant is ushered a warning the first time he or she fails to reach the line (within 2 m) and is eliminated following the second warning. Each participant’s score was tallied including the level and number of shuttles (20 m) reached before they were unable to keep up with the recording.

The 20 m shuttle run test was conducted by the doctoral student with the help of a gym teacher and it was performed in the gym under standardized conditions.

### 2.3. Questionnaires

Questionnaires were completed by all study participants. Prior to completion students were instructed to read the questions carefully and to devote sufficient time to answer the questions.

#### 2.3.1. Rosenberg Self-Esteem Scale

We used the Hungarian version of the Rosenberg Self-esteem Scale to measure students’ global self-worth by measuring both negative and positive feelings regarding the inner self. The 10-item scale is uni-dimensional. All items are answered using a 4-point Likert scale ranging from strongly agree to strongly disagree. Higher scores refer to higher self-esteem [42].

#### 2.3.2. EAT-26

The Eating Attitudes Test measures three aspects of eating behaviors. Cognitive restraint (CR) is a conscious effort by individuals to control what they eat to maintain or lose weight. Uncontrolled eating (UE) defines excessive eating in response to a loss of control over the food. Emotional eating (EE) is the need to overeat when an individual is unable to cope with emotionally negative situations and moods. The total value consists of tallying the scores regarding the three factors. Higher scores in the respective scales are indicative of greater cognitive restraint, uncontrolled, or emotional eating [23].

#### 2.3.3. BAT

The Body Attitude Test (BAT) is a self-report questionnaire including 20 items, scored on a 6-point Likert-scale. The test measures the subjective body experience and the attitudes toward the individual’s body, such as dissatisfaction with their own body, depersonalization of the body, complex feelings regarding overweight, lack of trust in one’s own body, hyperactivity and restlessness [43]. The cut-off score is 36. Higher scores reflect diminished levels regarding attitudes toward one’s own body.

The descriptives of the psychological variables are depicted in Table 1. The internal reliability of each scale reached a good level: Rosenberg Self-esteem Scale (Cronbach’s α = 0.88), The Body Attitude Test (Cronbach’s α = 0.83), The Eating Attitudes Test (Cronbach’s α = 0.81).

### 2.4. Data Analysis

Sample size and power analysis were performed for the overall population grouped by gender and BMI-for-age using power and sample size calculation program version 3.1.2 [44,45]. The sample size of *n* = 9 per group needed to detect a true difference of d = 6.017 in Rosenberg self-esteem with 95% power, where type I error probability is α = 0.05. Effect size analysis showed d = 0.631 (according to Cohen).

To analyze the psychological and physical variables we used Independent Samples *t*-tests, one-way ANOVA, Correlation analysis and Multivariate linear regressions after using the Kolmogorov–Smirnov test to check the normality of the data distribution. The normality test revealed a not significant result in all parameters (*p* > 0.05).

The Independent Samples *t*-tests were used to check the gender differences of the psychological variables.

Differences of BMI and shuttle run test (grouped by gender and BMI-for-age) as well as the psychological variables were evaluated by a one-way repeated ANOVA statistical test using Tamhane posthoc test.

Bivariate correlation analysis was performed calculating Spearman’s correlation coefficient (rho). Multiple regression analysis with various models considering the principle of multicollinearity was performed to reveal which factors could predict Self-esteem, BAT, Uncontrolled eating, Cognitive restraints, Emotional eating and shuttle run test. The correlation analysis showed significant associations of the psychological variables and some of the physical ones, more precisely: objective body shape (percentile zones) and subjective body shape (How do I see my body? This question refers to how somebody perceives his body shape, ranging from overweight/fat to skinny through a 5 point Likert-scale.).

The first regression model was performed to predict Self-esteem, BAT, Uncontrolled eating, Cognitive restraints, Emotional eating as the dependent variables regarding age, gender, Subjective body shape, BMI-for-age as independent variables.

The second linear regression and stepwise analyses of the data were performed to predict shuttle run test as the dependent variable regarding the Rosenberg Self-esteem Scale and Body Attitude Test values.

According to the Body Attitude Test results, we have divided the two gender groups into two groups: those who had worse attitudes toward their body (BAT-W), having more than 36 points, and those with better attitudes toward their body (BAT-B), having less than 36 points. Differences in these psychological variables between these girls and boys subgroups were evaluated by a one-way ANOVA statistical test.

## 3. Results

### 3.1. BMI-for-Age

**Figure 1:**
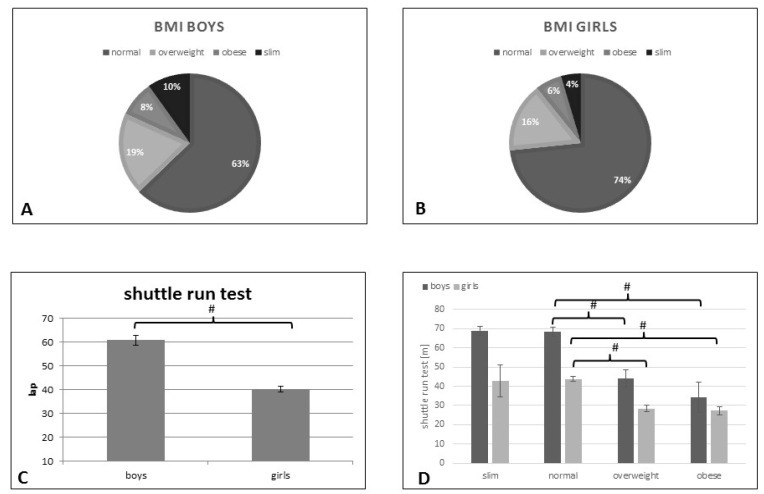
BMI-for-age in boys (**A**) and girls (**B**). Our results depicted significant differences in gender specific (**C**) and BMI-for-age specific shuttle run test (**D**) (data are shown as mean ± S.E.M, # = *p* < 0.001).

With respect to the boys, 10 percent were underweight, 63 percent normal weight, 19 percent overweight, and 8 percent obese (Figure 1A).

At the time of measurement 4 percent of the girls were underweight, 74 percent normal weight, 16 percent overweight, and 6 percent obese (Figure 1B).

### 3.2. The 20 m Shuttle Run Test

According to the 20 m shuttle run test data, 65% of boys (54 lap≥) and 59% of girls (38 lap≥) were in the Healthy Fitness Zone (HFZ). 35% of boys (≤45 lap) and 41% of girls (≤28 lap) were in the Needs Improvement zone (NIZ). The boys completed an average of 60.83 ± 25.79 laps. The girls accomplished 40.22 ± 16.34 laps. Students who meet the standards are classified as being in the Healthy Fitness Zone (HFZ), whereas students who fall below the standards are classified as being in Needs Improvement Zone (NIZ).

The analysis of the gender differences detected significantly lower shuttle-run test results among girls than when compared with boys (40.2 ± 1.13 vs. 60.8 ± 2.0; *p* < 0.001) (Figure 1C).

We observed no significant differences in shuttle run test results between slim and normal (68.81 ± 2.35 vs. 68.32 ± 2.46), as well as between overweight and obese boys (44.09 ± 4.65 vs. 34.4 ± 7.94), however, we discovered significantly lower performance in the obese and overweight boys than when compared with their classmates with normal BMI-for-age (Figure 1D).

The study results exhibited quite similar consequences in studying girls weight groups: there were no significant differences in shuttle run test outcomes between slim and normal nor between overweight and obese, however, we found significant differences between normal and overweight or obese girls’ aerobic capacity (42.78 ± 8.5 slim; 43.8 ± 1.28 normal; 28.44 ± 1.57 overweight; 27.23 ± 2.02 obese) (Figure 1C).

### 3.3. Psychological Tests Results

**Figure 2:**
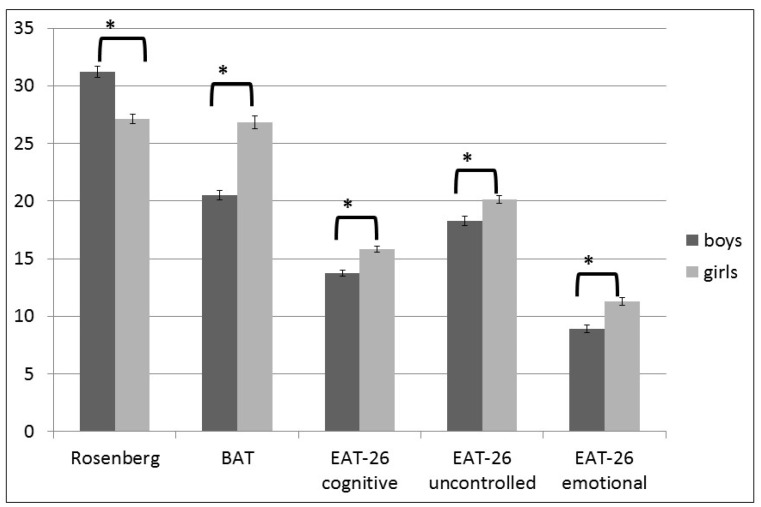
Our results showed significant differences in gender on the psychological tests (data are shown as mean ± S.E.M, * = *p* < 0.001). (Rosenberg = Rosenberg Self-esteem Scale; BAT = Body Attitude Test; EAT-26 = Eating Attitudes test: cognitive restraint, uncontrolled eating, emotional eating).

The study revealed a significant difference in self-esteem: Girls showed lower self-esteem than when compared with boys (*t* (374) = 6.62; *p* = 0.05). Girls also reported significantly negative body attitudes (*t* (374) = −9.12; *p* < 0.001) than when compared with boys and reached higher scores on each of the Eating attitudes subscales. Girls showed signs of uncontrolled eating (*t* (374) = −3.47; *p* = 0.01), cognitive restraint (*t* (374) = −5.56; *p* < 0.001) and emotional eating (*t* (374) = −4.78; *p* < 0.001) more often than when compared with secondary school-aged boys (Figure 2).

**Figure 3:**
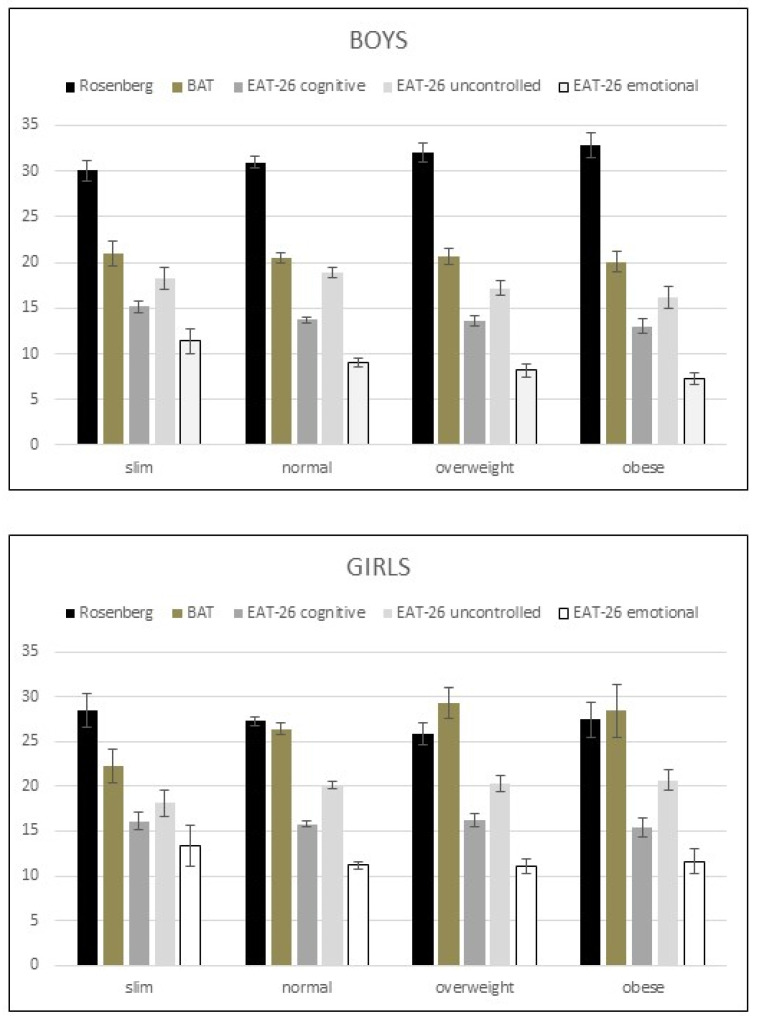
Psychological results in boys and girls showed no significant differences in the weight groups (data are shown as mean ± S.E.M, *p* < 0.05). (Rosenberg = Rosenberg Self-esteem Scale; BAT = Body Attitude Test; EAT-26 = Eating Attitudes test: cognitive restraint, uncontrolled eating, emotional eating).

Psychological tests did not reveal any significant differences in the comprehensive analyses of the weight groups in both boys and girls (Figure 3).

**Figure 4:**
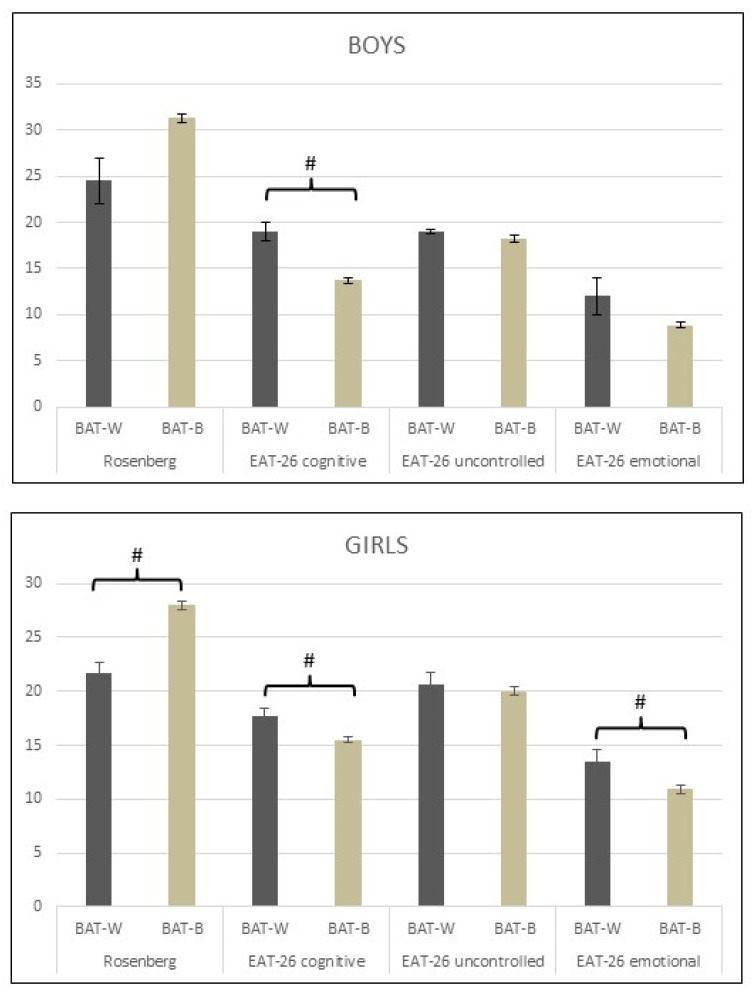
The analysis revealed significant differences between girls BAT-W (those with worse attitudes toward their body) and BAT-B (those with better attitudes toward their body) subgroups in Rosenberg Self-Esteem Scale and Eating attitudes cognitive restraints and emotional eating subscales. Additionally, our data showed significant differences between boys BAT-W and BAT-B subgroups in the cognitive restraints subscale of eating attitudes. (BAT-W: those with worse attitudes toward their body, BAT-B: those with better attitudes toward their body). #: significant difference, *p* < 0.05.

The ANOVA analysis detected significant differences between girls BAT-W and BAT-B subgroups in Rosenberg Self-esteem Scale (21.7 ± 0.95 vs. 27.97 ± 0.42; *p* < 0.001) and in the eating attitudes, such as cognitive restraints (17.8 ± 0.69 vs. 15.5 ± 0.27; *p* = 0.003) and emotional eating (13.5 ± 1.04 vs. 10.9 ± 0.37; *p* = 0.011). The results showed significant differences between boys BAT-W and BAT-B subgroups in eating attitudes cognitive restraints subscale (19.0 ± 1.0 vs. 13.7 ± 0.27; *p* = 0.03) (Figure 4).

The first multilinear regression analysis revealed that gender (b = −0.31, *t* = −6.41, *p* < 0.001) and subjective body shape predicted self-esteem (b = 0.16, *t* = 3.23, *p* = 0.001).

Body attitudes were also influenced by gender (b = 0.39, *t* = 8.71, *p* < 0.001) and subjective body shape (b = −0.32, *t* = −7.09, *p* < 0.001).

The Uncontrolled eating subscale of the Eating attitudes was only affected by gender (b = 0.18, *t* = 3.53, *p* < 0.001), just as the Emotional eating subscale (b = 0.23, *t* = 4.57, *p* < 0.001). In this case, however, BMI also played a weaker role (b = −0.12, *t* = −2.25, *p* < 0.05).

Cognitive restraint, similarly to self-esteem, showed strong associations with subjective body shape (b = −0.22, *t* = −4.32, *p* < 0.001) and gender (b = 0.26, *t* = 5.27, *p* < 0.001) (Table 2).

Second multivariate linear regression and stepwise analyses were performed to predict shuttle run test as the dependent variable regarding Rosenberg Self-esteem Scale and Body Attitude Test values. We revealed that both variables added statistically significantly to the prediction in boys F(2, 163) = 3.189, *p* = 0.044, *R*^2^ = 0.038.

## 4. Discussion

In our study lower self-esteem and higher scores for BAT and each scale of eating attitudes, such as Uncontrolled Eating (UE), Cognitive Restraints (CR) and Emotional Eat-ing (EE) were measured among adolescent girls compared to boys. Interestingly, not objective bodyweight but subjective body shape and gender predicted self-esteem and BAT scores and the cognitive restraints in the eating attitudes. Uncontrolled and emotional eating subscales were primarily influenced by gender, while BMI played only a weaker role. Additionally, self-esteem and body image were positively associated with cardiorespiratory performance in the boys, but not among girls.

The proportion of overweight and obese adolescents in our study group (Figure 1A,B) is slightly higher when compared to international data. A total of 107.7 million children were obese in 2015 worldwide [43], thus, obesity prevalence was as high as 5% among children, while overweight and obesity prevalence combined was as high as 23% [46,47]. Furthermore, overweight and obesity were more common in boys when compared to girls in our study (Figure 1A,B) which corresponds with international data [48].

In consideration of cardiorespiratory performance, measured by the shuttle run test, boys performed better than when compared with girls (Figure 1C) as it was anticipated, consistent with data in the literature [49]. Thin and normal weight adolescents performed significantly better on the cardiorespiratory test than when compared with their overweight or obese peers, among both genders (Figure 1D), of which findings are also congruent with previous studies [50,51]. Additionally, more girls have fallen into the Needs Improvement Zone than boys (41 vs. 35%).

Cardiorespiratory performance and its association with different psychological variables have been previously investigated in children and adolescents. For instance, Morales et al. in a Spanish study examining 1596 children, aged 8–11 years, found that cardiorespiratory fitness in girls and muscular fitness in boys closely associated with most of the dimensions of health related quality of life, including physical and psychological well-being, moods and emotions, self-perception, parent relation, home life and social acceptance [52]. In a study conducted in Australia, examining 821 elementary-school children, higher levels of cardiovascular fitness were found as a protective factor to body image concerns, regardless of the child’s body composition [53]. Interestingly, in accordance with our findings, cardiorespiratory performance was positively associated with self-esteem and body attitudes test results in our age-group (16.5 years) regarding adolescent boys, however, not in girls.

A relationship focusing on overweight, obesity and low self-esteem has been investigated several times and the results varied. In our findings, self-esteem was predicted by subjective body shape instead of objective bodyweight. Several studies depict lower self esteem among obese adolescents [3,54], while others found associations only among subgroups based on age or race [55,56] or for a specific domain of self-esteem [57]. A literature review indicated it was unclear whether self-esteem was consistently related to obesity [58]. There may be some other factors that mediate the relationship between obesity and psychological problems. According to earlier studies, body-image satisfaction may be the intermediate factor between obesity and self-esteem [59].

Assessing body image satisfaction, the Body Attitude Test (BAT) was used, which was originally designed for patients, primarily women suffering from eating disorders [43]. In the current study, girls scored higher scores on BAT than boys that could mean that boys have more positive attitudes toward their bodies, but it is also possible that the questionnaire does not measure enough aspects of male body attitudes. Later, Probst et al. evaluated the psychometric properties of body image questionnaires including BAT among non-clinical female and male subjects and found that these tests can reliably differentiate between the two sexes and those individuals who are and who are not suffering from body image concerns [60].

In our study, no significant correlation was found among the actual weight status and body image satisfaction, neither the eating attitude subscales (Figure 3). According to the literature, there are many factors influencing body image and eating attitudes. Studies have shown that social media consistently and overly represent idealized body types which potentially contribute to poor body image and disordered eating behaviors which may be underpinned by the beauty and diet industries [61,62,63]. Adolescent body image is also heavily influenced by family and social relationships. Adolescents who are exposed to negative body and diet conversations with friends and family members have higher levels of body dissatisfaction and higher BMIs and are manifested in unhealthy eating behaviors later in life [64,65].

In our study, positive body image was associated with higher self-esteem and lower cognitive restraints and emotional eating in the eating attitude subscales in girls and with lower cognitive restraints in boys (Figure 4). Previous studies indicated that positive body image has been associated with lower concerns regarding appearance, healthier BMI, increased physical activity and better general health habits in adolescents [66,67,68,69]. On the other hand, body dissatisfaction may trigger emotional eating [70,71] which has been associated with greater adiposity and higher BMI [71,72]. Furthermore, emotional eaters tend to turn to high-energy and low-nutrient foods in response to their emotional feelings [73]. Restrained eaters take control over what they eat and when they eat, however, paradoxically, under certain conditions they lose control, for example during negative mood states [74].

Additionally, in our study, all aspects regarding eating attitudes were affected by gender. Girls were more prone to emotional and uncontrolled eating and cognitive restraints, when compared to boys and BMI, had only a weaker role in emotional and uncontrolled eating. It is well-known that eating disorders are more common in women than in men and adolescent girls are at high risk. Girls experience more food-related conflicts than when compared with boys and they also experience more dissatisfaction with their body that may affect weight regulation [75].

Finally, there may be some gender relevant analysis issues with the use of the EAT-26 questionnaire which was originally validated and used in female populations [23]. However, in a recent study, Schaefer et al. have demonstrated that no items of EAT-26 met the criteria for statistically significant differential item functioning suggesting that the EAT-26 questionnaire operates similarly among males and females [76]. Although EAT-26 seems to be an appropriate measure to assess thinness-oriented eating disorder symptoms in non-clinical samples of men, it may not evaluate symptoms that are more specific in the male population (e.g., muscularity-oriented concerns) [77].

## 5. Limitations

There are some limitations of the current study that could be addressed in future research. First, the self-esteem of the study participants was measured by the Rosenberg Self-esteem Scale. However, the scale is widely used to measure global self-esteem, there are more theories that describe self-esteem as a more complex phenomenon. For further research, it would be interesting to compare the associations of different aspects of self-esteem to the variables used in the current study. Second, BAT and EAT-26 questionnaires were originally designed and validated for female subjects. Although these tests have already been used in male populations, they may not measure male specific items properly, and therefore, the results must be interpreted carefully. Third, the relatively small number of participants in the overweight and obese subgroups also warns us to interpret the associations with caution. These limitations should be taken into account and eliminated when planning future studies.

## 6. Conclusions

Based on our results we want to emphasize that attention must be focused on both psychological and physical health during adolescence. Although overweight and obesity were more commonly seen in adolescent boys, girls were more prone to have lower levels of self-esteem, poorer body images and experienced increased problems regarding eating behavior. Furthermore, no significant correlation was found between BMI and psychological test results in either boys or girls. However, cardiorespiratory performance was positively associated with self-esteem and body image among boys, and it had a negative correlation with body weight in both genders. Since adolescent health behaviors can predict adult health status [1,78], future research might require a longitudinal follow-up to track changes in attitudes of adolescents regarding their own body, self-esteem and eating habits and examine other potential mediators among physical and psychological variables, such as the role of social and family influence.

## Figures and Tables

**Table 1:** Descriptives regarding the psychological variables.

Psychological Variable	Mean	SD	Minimum	Maximum	Skewness/Kurtosis
Self-esteem (RSES)	28.91	6.32	12	40	−0.251/−0.471
Body attitudes (BAT)	24.02	7.70	13.13	55	0.865/0.960
Uncontrolled eating (EAT-UE)	19.29	5.18	9	33	0.339/−0.391
Cognitive restraints (EAT-CR)	14.92	3.74	6	26	0.080/−0.352
Emotional eating (EAT-EE)	10.24	4.98	6	24	0.598/0.725

**Table 2:** Results of the regression analysis: standardized *β* and *R*^2^ values. (SBSH: Subjective body shape). * *p* < 0.05, ** *p* < 0.001.

Dependent Variables	Predictors				
	Age	Gender	SBSH	BMI for Age	*R* ^2^
Self-esteem (RSES)	0.09	−0.31 **	0.16 **	0.07	0.16 **
Body attitudes (BAT)	0.00	0.39 **	−0.32 **	0.01	0.28 **
Uncontrolled eating (UE)	−0.06	0.18	0.06	−0.02	0.04 *
Cognitive restraints (CR)	0.02	0.26 **	−0.22 **	−0.09	0.12 **
Emotional eating (EE)	−0.01	0.23 **	−0.07	−0.12*	0.07 **

## Data Availability

The data presented in this study are available on request from the corresponding author.
